# Urinary Phthalate Concentrations in Mothers and Their Children in Ireland: Results of the DEMOCOPHES Human Biomonitoring Study

**DOI:** 10.3390/ijerph14121456

**Published:** 2017-11-25

**Authors:** Elizabeth Cullen, David Evans, Chris Griffin, Padraig Burke, Rory Mannion, Damien Burns, Andrew Flanagan, Ann Kellegher, Greet Schoeters, Eva Govarts, Pierre Biot, Ludwine Casteleyn, Argelia Castaño, Marike Kolossa-Gehring, Marta Esteban, Gerda Schwedler, Holger M. Koch, Jürgen Angerer, Lisbeth E. Knudsen, Reinhard Joas, Anke Joas, Birgit Dumez, Ovnair Sepai, Karen Exley, Dominique Aerts

**Affiliations:** 1 Department of Community of Health, Health Service Executive, St. Mary’s, Naas, Kildare W91 NR29, Ireland; 2 Department of Public Health, Health Service Executive, Galway H91973, Ireland; david.evans@hse.ie; 3 Public Analyst’s Laboratory, Health Service Executive, Dublin D02 P667, Ireland; chris.griffin@hse.ie; 4 Public Analyst’s Laboratory, Health Service Executive, Galway H91 Y952, Ireland; padraig.burke@hse.ie (P.B.); rory.mannion@hse.ie (R.M.); andrew.flanagan@hse.ie (A.F.); 5 Project Manager, Health Service Executive, Palmerstown, Dublin D20 X318, Ireland; damien.burns@hse.ie; 6 Environmental Health Service, Health Service Executive, Carrick on Shannon, Co Leitrim N41 XC59, Ireland; ann.kellegher@hse.ie; 7 Flemish Institute for Technological Research (VITO), Sustainable Health Mol B-2400, Belgium; greet.schoeters@vito.be (G.S.); eva.govarts@vito.be (E.G.); 8 Biomedical Department, University of Antwerp, B-2000 Antwerp, Belgium; 9 Federal Public Service Health, Food Chain Safety and Environment, Brussels 1060, Belgium; pierre.biot@environnement.belgique.be; 10 Center for Human Genetics, University of Leuven, Herestraat 49, 3000 Leuven, Belgium; ludwine.casteleyn@empreva.belgie.be (L.C.); dumez.birgit@gmail.com (B.D.); 11 Carlos 111 Institute of Health, National Centre for Environmental Health, Majadahonda, Madrid 28220, Spain; castano@isciii.es (A.C.); m.esteban@isciii.es (M.E.); 12 German Environment Agency, Berlin 14195, Germany; marike.kolossa@uba.de (M.K.-G.); Gerda.Schwedler@uba.de (G.S.); 13 Institute for Prevention and Occupational Medicine of the German Social Accident Insurance, Institute of the Ruhr-Universität Bochum (IPA), Bochum 44789, Germany; koch@ipa-dguv.de (H.M.K.); angerer@ipa-dguv.de (J.A.); 14 Department of Public Health, University of Copenhagen, Copenhagen 1353, Denmark; liek@sund.ku.dk; 15 BiPRO GmbH, Munich 81545, Germany; reinhard.joas@bipro.de (R.J.); anke.joas@bipro.de (A.J.); 16 Centre for Radiation, Chemical and Environmental Hazards, Public Health England, Chilton OX11 ORQ, Oxfordshire OX11 ORQ, UK; ovnair.sepai@phe.gov.uk (O.S.); Karen.Exley@phe.gov.uk (K.E.)

**Keywords:** phthalates, human biomonitoring, exposure, endocrine disruptors

## Abstract

*Background*: Phthalates are chemicals which are widespread in the environment. Although the impacts on health of such exposure are unclear, there is evidence of a possible impact on the incidence of a diverse range of diseases. Monitoring of human exposure to phthalates is therefore important. This study aimed to determine the extent of phthalate exposure among mothers and their children in both rural and urban areas in Ireland, and to identify factors associated with elevated concentrations. It formed part of the ‘Demonstration of a study to Co-ordinate and Perform Human Biomonitoring on a European Scale’ (DEMOCOPHES) pilot biomonitoring study. *Methods*: the concentration of phthalate metabolites were determined from a convenience sample of 120 mother/child pairs. The median age of the children was 8 years. A questionnaire was used to collect information regarding lifestyle and environmental conditions of the children and mothers. Rigorous quality assurance within DEMOCOPHES guaranteed the accuracy and international comparability of results. *Results*: Phthalate metabolites were detected in all of the samples from both children and mothers. Concentrations were significantly higher in respondents from families with lower educational attainment and in those exposed to such items as polyvinyl chloride (PVC), fast food and personal care products (PCP). *Conclusions*: The study demonstrates that human biomonitoring for assessing exposure to phthalates can be undertaken in Ireland and that the exposure of the population is widespread. Further work will be necessary before the consequences of this exposure are understood.

## 1. Introduction

Phthalates are a group of chemicals found in many consumer goods, including personal care products (PCPs), cosmetics, plastic products, children’s toys, household furnishings and consumption of convenience and fast foods such as ready prepared meals and takeaways. Phthalates provide flexibility to rigid materials such as polyvinyl chloride (PVC) and they can also act as a lubricant [1]. In addition, phthalates may be present in food as a result of migration from food packaging [2,3].They are not chemically bound to the products to which they are added and can be released to the environment during use or disposal. The widespread use of phthalates in daily life results in exposure from direct contact with products containing phthalates, from consuming food which has been in contact with phthalate containing packaging material and by inhalation of contaminated air. Phthalate diesters are rapidly metabolized, initially by substituting one of the side-chains with glucuronate. Longer hydrocarbon side-chains may be oxidized to increase solubility. These are quickly excreted and do not accumulate in the body.

The impact of phthalate exposure to human health is unclear [4]. There is concern that some phthalates may act as endocrine disrupting compounds, leading to adverse reproductive and developmental effects [5]. Studies have shown significant impacts on laboratory animals but due to inadequate data, the human health impacts from low dose exposure are difficult to determine [6]. Nevertheless, some studies have shown that phthalate exposure adversely affects human male reproductive development [7] and sperm quality [8,9], and is also associated with risk factors for cancer, asthma and allergies [10]. Phthalates have been associated with obesity [11] but their impact on overall cardiovascular health is unclear [12]. As a result, there is increasing public health concern of the general population’s exposure to phthalates [4].

Due to their widespread use and the lack of clarity in terms of effects, it is important that exposure concentrations are monitored. There is a relative absence of data on exposure to phthalates in Ireland. Human biomonitoring (HBM) is a tool by which concentrations of exposure of humans to environmental contaminants may be assessed. This study formed part of the DEMOCOPHES pilot study across 17 European Union countries [1,13] and which involved monitoring of four key environmental pollutants (mercury, cadmium, cotinine and phthalates), in 1844 mother/child pairs. The aim of the present study was to determine the concentrations of seven biomarkers, metabolites from five phthalates, in urine samples from mothers and their children in Ireland. The metabolites are arranged in order of ascending molecular weight (Table 1).

## 2. Materials and Methods

### 2.1. Sampling 

A convenience sample of eight schools (four urban and four rural) participated in the study. Schools in urban and rural locations were contacted by letter by Environmental Health Officers (EHOs), describing the project and inviting their participation in the study. Urban and rural locations were determined by population density data from the Central Statistics Office, in Dublin. Larger schools were chosen in order to maximize the response rate. Following receipt of consent from the school, parent information packs and an invitation letter were given to all children aged 6 to 11 years attending the school. The criteria for inclusion were that participants should not be in hospitals, institutions or homeless, and be in good health without any metabolic or renal condition. In addition, mothers had to be aged 45 years or less, to have lived in the area for at least five years, and to be able to speak English well. Of the invitation letters (1185 urban and 650 rural), replies were received from 551 families (30% response rate; 20% urban and 50% rural). Of these, 311 were positive (142 urban and 169 rural; 12% and 26% of invitation letters, respectively). A further 33 families were excluded during quota sampling as they did not meet the eligibility criteria. Following quota sampling, 120 mother / child pairs, (60 urban and 60 rural) were subsequently included in the study (6.5% of the families contacted). 120 mother/child pairs was the standardised number of respondents for all countries participating in the DEMOCOPHES study).

Appointments were made with the mothers who had agreed to participate, for a home visit to take place at a time that suited the mother and child. Following a home visit from the Environmental Health Officer (EHO) to obtain consent, the EHO subsequently visited the selected families in their homes from October 2011 to January 2012 to administer a questionnaire. The questionnaire was performed by the EHO as an interviewer guided questionnaire with the mother. The aim of the questionnaire was to obtain socio-demographic details and information on both diet and possible exposure to phthalate containing products. Sociodemographic profile variables included highest education concentration in family (primary or lower secondary, higher secondary, third concentration), marital status (single), age of mother (<35, 35–40, >40) and child (5–8, 9–11), gender of child, smoking characteristics of household (mothers who smoke, households with at least one smoker), employment outside home (mothers and fathers). Information in terms of dietary and other possible sources of phthalate exposure included consumption (several times a week or once a week or less) of meat, convenience or fast food and chewing gum; time spent in a new car (at least one hour a day, or one hour a day or less), wearing plastic gloves (daily, less than daily, never), using personal care products (high, moderate, low), PVC in house (in floors and walls, in floors or walls, no PVC), and area of residence (urban or rural). The questionnaire took approximately forty five minutes to complete and the mean duration of the home visit was two hours and fifteen minutes. Early morning urine samples were taken from mother and child at this time. The urine samples were collected from the home in calibrated cool-boxes and transported to the survey office where they were stored in a designated fridge at temperatures between 4–8°C. Samples were stored for a maximum of five days and were transported in cool-boxes to the laboratory at the end of each week. A gift voucher was given to the mother as a gratuity. Quality audits were carried out in each survey office by another fieldworker to ensure consistency and that a standardized approach was adopted during the fieldwork.

### 2.2. Chemical Analysis 

Urine samples were analysed by the Public Analyst Laboratory (Health Service Executive, Dublin, Ireland), employing a validated procedure. Quality standards were assured by successfully completing three proficiency tests (a requirement of the DEMOCOPHES project) [1] The limits of detection (LOD) were between 0.015 to 0.33 µg/L, and the limits of quantification (LOQ) were between 0.05 to 1 µg/L. Isotopically labelled internal standards were added to an aliquot of urine which was subjected to enzymatic hydrolysis at 40°C for two hours. Sample clean-up and analyte enrichment was carried out on-line using a Restricted Access Material and back flushed onto an analytical column for chromatographic separation. Detection was by tandem mass spectrometry with positive electrospray ionisation. To allow for differences in urinary concentrations, the results are presented per gram of creatinine. 

### 2.3. Statistical Analysis 

As data for phthalate metabolites were not normally distributed, Mann Whitney U tests were undertaken to determine the significance of differences between biomarker concentrations in mothers and children. One-way Anova (on log transformed data) was undertaken to establish the significance of differences by sociodemographic factors and other factors associated with exposure. Data analysis was undertaken using SPSS Statistics v.23 (IBM Inc., Armonk, NY, USA). Ethical approval was obtained from the Faculty of Public Health Medicine (Royal College of Physicians of Ireland, 23 September 2011). 

## 3. Results 

### 3.1. Profile 

The socio-demographic profile of the respondents is outlined (Table 2). The average age of mothers was 38.1 years (median = 39.0) and 53% (*n* = 63) had received third-concentration education. There was a smoker in 43% of households, with 29% (*n* = 35) of mothers smoking. 

Most of the fathers (79%) and over a half of the mothers (59%) worked outside the home. The average age of children was 8.5 years (median = 8.0) with a larger proportion of boys (53%, *n* = 63).

### 3.2. Overall Phthalate Exposure

All seven phthalate metabolites were detected in all of the Irish children’s samples and in addition, five of the seven metabolites were detected in all of the Irish mothers’ samples with the remaining two, MEHP and MBzP detected in 90% and 97.5% of samples respectively (Table 3).

The two phthalates with the highest measured geometric mean concentration in children were MiBP, followed by MEP; while in mothers, the opposite occurred, with highest concentration being MEP, followed by MiBP. For MEP, geometric mean concentrations were higher in mothers than children. This association was statistically significant (*p* < 0.05). The geometric mean of urinary concentrations of six of the seven phthalate metabolites was higher in Irish children than in their mothers. With the exception of MEHP, these higher concentrations were statistically significant (*p* < 0.05). 

### 3.3. Sociodemographic Patterns

Age was a factor in determining exposure in children to MBzP and MnBP, with higher concentrations of these compounds found in children aged 5–8 years, when compared to children aged 9–11 years (*p* = 0.04 and 0.03) respectively. Higher concentrations of MEOHP were also found in children living in urban areas when compared to rural areas (*p* = 0.03). The only sociodemographic variable associated with exposure in both mothers and children was education, where higher concentrations of MiBP were inversely related to educational attainment. Higher concentrations were found in mothers who had just completed education to primary concentration in contrast to mothers who had completed third-concentration education (*p* = 0.006), this association was also noted in their children (*p* = 0.002). There were no other significant sociodemographic variables associated with exposure concentrations (Table 4).

### 3.4. Other Factors Associated with Exposure

All statistically significant associations between phthalate concentrations and environmental exposures in the Irish children and their mothers are shown (Table 5). MiBP, the compound with the highest geometric mean in children at 41.4 µg/g creatinine, was significantly associated with exposure to PVC in the house, (*p* = 0.007). Concentrations of MEP, the compound with the highest recorded value in mothers at 50.2 µg/g creatinine and second highest in children, was associated with the use of personal care products by Irish children (*p* = 0.02) and with the consumption of convenience foods in the last 24 h by mothers (*p* = 0.02). There was also an inverse association between the consumption of meat and concentrations of MEP in mothers (*p* = 0.02). 

Concentrations of MEOHP, MEHHP and MEHP (all metabolites of DEHP) in Irish children were significantly associated with the use of chewing gum. In Irish mothers, concentrations of MiBP were significantly higher in those who consumed fast food more than once a week (*p* = 0.02). In addition, significantly higher concentrations of MnBP were found in mothers who wore plastic gloves (*p* = 0.03). An inverse association was noted in children between the concentrations of MBzP and the consumption of cheese (*p* = 0.007).

## 4. Discussion

This is the first study to examine the exposure of the Irish population to phthalates. It is clear that exposure to phthalates is widespread, with detectable concentrations of all seven compounds found in samples from all the children. Five of the seven compounds were detected in all the mothers, with MBzP in 97.5% and MEP in 90%. The concentrations detected in Irish mothers and children are comparable, but slightly higher for some compounds than the concentrations found in the overall DEMOCOPHES cohort [13]. A similarly high pattern of exposure was found in Spain [14] where phthalates were detected in all the mother and child participants, and in Germany, where all of the phthalate compounds were detected in all of the participants apart from MEHP which was detected in 93% of children and 85% of mothers [15]. Similarly, a population based survey in Sweden found that all of the children and mothers had detectable concentrations of phthalate compounds with the exception of MEHP, which was found in 98% of mothers [16]. Such high exposure patterns are not surprising as phthalates have been described as being “ubiquitous” in the environment [5]. 

The geometric mean concentrations of the Irish children were substantially higher than those found in their mothers, with the exception of MEP. Similarly, the geometric mean concentrations of the DEMOCOPHES children were much higher than their mothers, with the exception again being MEP [13]. A similar pattern was also noted in Spain [14] in Sweden [16] and in the USA where, apart from MEP, all of the compounds were detected at lower concentrations in mothers than in their children [17]. MEP is a common constituent of cosmetic and personal care products and it is not surprising that higher concentrations of MEP metabolites should be found in mothers. Younger children in Ireland (aged 5–8 years) were at higher risk than older children (aged 9–11 years) for all compounds, apart from MEP (and MEHP which was the same concentration in both age groups). Higher concentrations of phthalate compounds were also found in younger children in Germany when compared to older children [18], and in the UK [19]. A similar pattern was also noted in the DEMOCOPHES cohort, where younger children again had higher concentrations of all compounds apart from MEP [13]. The phthalates with the two highest geometric mean concentrations were MiBP followed by MEP. This is in contrast to adults where the highest concentration was MEP, followed by MiBP. There is a lack of a consistent pattern for concentrations of MiBP and MEP in other studies. Studies of children in the UK [19], Germany [15], Austria [20], and the overall DEMOCOPHES cohort found higher concentrations for MiBP in children than MEP [13]. In contrast, other studies from Austria [21] and the USA [17] found higher concentrations of MEP. Such variations may be indicative of the varying exposure of populations to phthalate compounds in general and reflects both the timing of the study and also country specific differences such as life style and product use [22].

Higher concentrations of phthalates in children in general have been attributed to their relative body size, their higher hand to mouth behaviour, more time spent on the floor, contact with toys and by differences in metabolism [23]. These results suggest that there may be a need for stricter regulation for products specifically designed for children, such as controls on phthalates classified as endocrine disruptors. In this regard, the recent proposal by the EU to place restrictions on the phthalate compounds DEHP, DBP, DiBP and BBP is welcome [24].

In relation to sociodemographic variables, concentrations of the compound MEOHP were higher in Irish children who were from an urban background. This compound is a metabolite of DEHP, and is frequently added to plastics to make them flexible; it is therefore found in many household products such as table cloths, rainwear and shower curtains. It is difficult to know why concentrations of this compound should be higher in urban communities; this finding was not evident in children in the overall DEMOCOPHES results [13]. Urban residence has however been associated with higher concentrations of MiBP in mothers and children in Germany [15], and also in Egyptian women and this has been attributed to lifestyle and behavioral factors [25]. In contrast concentrations MiBP and also MEP were higher in Spanish children from rural areas and this was ascribed to either lifestyle or environmental factors in their location 14]. Higher concentrations of some phthalate compounds have also been noted in mothers and children living in rural areas and this has been attributed to an increased exposure to PVC in these areas [16]. Both MiBP and MnBP in Chinese children have also been associated with urban living and this has been attributed to run-off from electrical equipment in the heavily industrialized area where the study was undertaken [26]. Studies in the Czech Republic, Hungary and Slovakia have not detected an impact of rural versus urban living on concentrations of this compound [27]. No difference was also noted between the concentrations of phthalate metabolites in urban and rural mothers and children in a Danish study [28]. The varying patterns of phthalate exposure noted with respect to location are indicative of the varying exposure of populations to phthalate compounds in general and reflect the ubiquitous nature of the product.

The only other sociodemographic variable that was correlated with exposure was educational attainment, where concentrations of MiBP were lower in both Irish mothers and their children as educational achievement in the household increased. Similarly, increased educational attainment in the household was also associated with lower concentrations of the DEHP metabolites, MEOHP, MEHHP and MEHP in the DEMOCOPHES cohort. Educational attainment has been related to perceived concern about the dangers of chemicals in the environment as well as being proactive in engaging in health behaviour to reduce exposure [4,29]. 

MEP and MiBP were the compounds that showed the highest concentration in both children and mothers. Exposure to household products such as perfumes, colognes, deodorants, soaps, shampoos and hand lotions will result in the excretion of MEP. Concentrations of MEP in Irish children were correlated with use of personal care products. Higher concentrations of MEP have recently been associated with obesity [11]; and this may have also been a contributing factor as childhood obesity is a significant issue in Ireland [30]. This association would require further investigation as body mass index was not calculated in the current study. Surprisingly, concentrations of MEP in Irish mothers were not associated with the use of personal care products. Irish women use cosmetics, as evidenced by national beauty and personal care market figures which estimate that the market was valued at €988 million for 2011 and is due to grow by 3% over the year [31]. It is therefore difficult to account for this finding. In contrast, concentrations of MEP were associated with use of personal care products in both mothers and children in the DEMOCOPHES cohort, in mothers in Spain who used body lotions and creams, and in children in Spain who used fragranced products [14] and again in users of personal care products in the Czech Republic, Hungary and Slovakia [27]. MEP concentrations were also associated with the use of fragrances and make-up in German mothers [15] and with eye makeup and sunscreen in Swedish mothers [16]. Although exposure to personal care products was only associated with concentrations of MEP in Irish children, concentrations of this compound in Irish mothers were linked with the consumption of convenience foods in the last 24 h. Likewise, the consumption of fast foods was associated with MiBP in Irish mothers, but not in their children. Such foods are often wrapped in plastic. Fast food has been identified as a possible source of exposure to DEHP and DiNP in the U.S. [17] and MBzP was associated with increased consumption of fast food in Spain [14]. Exposure to PVC was the only factor associated with concentrations of MiBP in Irish children. Similarly, PVC exposure in children was associated with MBzP and DEHP metabolites in Germany [15], and also with MnBP and MBzP in Spanish children [14]. PVC is the most common product that contains DiBP, the precursor for MiBP. In the European DEMOCOPHES study exposure to PVC was associated not only with MiBP concentrations, but also with MBzP concentrations in mothers and children and MnBP in mothers [13]. MEHP, MEHHP and MEOHP concentrations, the metabolites of DEHP, were associated with chewing gum in Irish children. This finding is similar to those of the DEMOCOPHES cohort which also found an association between chewing gum use and MEHHP and MEOHP [13]. No such association between chewing gum use and phthalate exposure was found in Irish mothers. An association between chewing gum and phthalates has been attributed to chewing gum being a proxy for foods that are processed, flavoured and packaged [13]. DEHP (the precursor of MEHP, MEHHP and MEOHP) was identified by the EU in February 2017 as an endocrine disrupting chemical which may damage fertility and the unborn child [32]. MnBP excretion will result from exposure to DBP. DBP is found in many products including paints, hair spray and nail polish. MnBP concentrations in Irish women were associated with the use of plastic gloves and in the DEMOCOPHES study with exposure to PVC and personal care products. Interestingly, while no association was noted between DEHP metabolites and the use of PCPs in Irish mothers, their use was inversely related with the use of PCP products in the DEMOCPHES cohort [13]. 

The health significance of the findings of this study is unclear. Work has been undertaken on reference values of specified chemicals by the German Human Biomonitoring Commission, and is based on the 95% confidence interval of the 95th percentile of the concentration of a chemical substance in a population. Such reference values cannot be used to assess health risk [33]. The concentrations of MBzP, MiBP and MnBP in both Irish children and their mothers were below such reference concentrations established [34]. In relation to the sum of the metabolites 5OH-MEHP and 5oxo-MEHP the German Human Biomonitoring Commission published two guidance values, HBM –I and HBM II. HBM I values are those values below which there is no risk for human health. HBM II concentrations are concentrations above which there is an increased risk for adverse effects. The HBM I value of these metabolites was 500 μg/L in children and 300 μg/L in adults. No concentrations in the Irish children were above HBM concentrations II, in contrast to 1.7% of the children in the German study. A smaller number, 0.02% of mothers exceeded the lower threshold of 300 μg/L for adults in Ireland. However, studies of the total diet in Ireland have shown that the overall exposure of the Irish population to phthalates from food is low in both adult and child population groups; average, as well as above average exposure to phthalates was found to be well below the respective Tolerable Daily Intake set by the European Food Safety Authority [35]. However, in view of the concerns regarding the links of phthalates with cancer, reproductive toxicity, asthma and other illnesses, caution would seem to be the best policy until further information is available. In addition, continuous human biomonitoring will be necessary to track changes in exposure to pollutants over time [15]. In that regard, the assessment of phthalate exposure of people who develop cancer and who are entered in the Irish National Cancer Registry would also be of interest.

The supply and use of certain phthalates are restricted by various pieces of EU legislation. Regulation 1907/2006 [36] or REACH (Registration, Evaluation, Authorisation and Restriction of Chemicals) aims to improve the protection of human health and the environment from risks that can be posed by chemicals while enhancing the competitiveness of the EU chemicals industry. Regulation 10/2011 [37] controls phthalate use in food contact materials, DEHP, BzBP, DBP, DiNP and DiDP are the only phthalates permitted as plasticisers. DBP, DiBP and BzBP are prohibited in Cosmetics by Regulation 1223/2009 [38]. Toy safety is controlled by Directive 2009/48/EC [39] which does not mention phthalates, but toys containing substances of very high concern (SVHC) above 0.1% are not compliant with REACH. 

There are some issues that need to be kept in mind when interpreting this data; phthalates have a relatively short half-life in the body and the concentrations obtained in this study represent exposure over the previous 12 h. Exposure to products that may not occur on a daily basis may not result in high biomarker concentrations. Analysis of the differences in urinary concentrations by demographic factors and factors associated with exposure was undertaken using univariate analysis (One-way Anova). Further insight in terms of the relative importance of each predictor would have been achieved if multivariate techniques such as multiple regression were employed. In addition, the sample is not representative of the age and sex composition of the Irish population, ideally repeated testing on larger samples would be needed at regular intervals using larger, representative samples as in NHANES studies [40] , in order to more fully comprehend the impacts of exposures to phthalate sources on biological samples. Association of such work with disease registries would also be useful.

## 5. Conclusions

Exposure to phthalates is extensive and from multiple sources. All seven phthalate compounds were detected in all of the children’s samples and five of the seven compounds were found in all of the mother’s samples. The urinary concentrations of the majority of the phthalate metabolites were higher in children than in their mothers. Exposure to chewing gum, PVC and PCP in Ireland were associated with higher concentrations in children, and consumption of fast foods and convenience foods were a significant factor in mothers. Human Biomonitoring is a useful tool for assessing overall exposure from multiple diverse sources, however further work is needed in order to more fully understand the determinants of distribution of these compounds and their impact on health. A copy of the questionnaire could be found in the supplementary material.

## Figures and Tables

**Table 1:** Metabolites and the parent compounds assessed.

Metabolite	Parent Compound
Monoethyl phthalate (MEP)	Diethyl phthalate (DEP)
Monobutyl phthalate (MnBP)	Dibutyl phthalate (DBP)
Monoisobutyl phthalate (MiBP)	Diisobutyl phthalate (DiBP)
Monobenzyl phthalate (MBzP)	Benzylbutyl phthalate (BBP)
Mono-2-ethylhexyl phthalate (MEHP)	Di-2-ethyhexyl phthalate (DEHP)
Mono-(2-ethyl-5-hydroxyhexyl) phthalate (MEHHP)	
Mono-(2-ethyl-5-oxohexyl) phthalate (MEOHP)	

**Table 2:** Sociodemographic profile of respondents.

Profile	No.	%
**Area of residence**		
Urban	60	50.0
Rural	60	50.0
**Highest educational concentration in family**	8	6.7
Primary or lower secondary	49	40.8
Higher secondary or post-secondary, non-third concentration		
Third-concentration education	63	52.5
**Marital status**		
Single mothers	16	13.3
**Age of mothers**		
<35 years	34	28.3
35–40 years	41	34.2
>40 years	45	37.5
**Age of child (years)**		
5–8	61	50.8
9–11	59	49.2
**Gender of child**		
Boy	63	52.5
Girl	57	47.5
**Smoking characteristics**		
Mothers who smoke	35	29.2
Households with at least one smoker	51	42.5
**Working outside the home**		
Mothers	71	59.2
Fathers	82	78.8

**Table 3:** Exposure of mothers and children to phthalates µg/g (creatinine adjusted).

Phthalate	Mothers	Children	Mann Whitney U test
**MEP**			6006.5 (mean rank mother = 130.5, child = 110.6) *p* = 0.026
Percent above LoQ	100.0	100.0	
Geometric mean	50.2	38.7	
Arithmetic mean	88.0	53.6	
Minimum/Maximum	6.0/725.0	4.4/462.1	
**MnBP**			5773.5 (mean rank mother = 108.6, child = 132.4) *p* = 0.008
Percent above LoQ	100.0	100.0	
Geometric mean	18.5	26.1	
Arithmetic mean	22.8	29.8	
Minimum/Maximum	3.32/159.3	10.1/511.4	
**MiBP**			4722.5 (mean rank mother = 99.9, child = 141.2) *p* < 0.001
Percent above LoQ	100.0	100.0	
Geometric mean	23.8	41.4	
Arithmetic mean	31.0	55.7	
Minimum/Maximum	5.1/181.2	7.4/91.5	
**MBzP**			5235.5 (mean rank mother = 104.1, child = 136.9) *p* < 0.001
Percent above LoQ	97.5	100.0	
Geometric mean	3.1	5.4	
Arithmetic mean	5.1	8.2	
Minimum/Maximum	0.4/50.4	1.0/65.9	
**MEHP**			6831.5 (mean rank mother = 117.4, child = 123.6) *p* = 0.493
Percent above LoQ	90.0	100.0	
Geometric mean	2.8	3.5	
Arithmetic mean	4.1	4.5	
Minimum/Maximum	0.5/28.8	1.15/20.5	
**MEHHP**			4281.0 (mean rank mother = 96.2, child = 14.8) *p* < 0.001
Percent above LoQ	100.0	100.0	
Geometric mean	17.0	32.8	
Arithmetic mean	23.1	39.8	
Minimum/Maximum	4.2/336.5	12.4/284.8	
**MEOHP**			4139.5 (mean rank mother = 95.0, child = 146.0) *p* < 0.001
Percent above LoQ	100.0	100.0	
Geometric mean	8.8	17.7	
Arithmetic mean	11.7	21.6	
Minimum/Maximum	2.0/125.1	6.3/157.1	

**Table 4:** Association between phthalate exposure and sociodemographic variables *.

Compound	Irish Children	Irish Mothers
**MnBP**	Younger > older *p* = 0.03	*p* > 0.05
**MiBP**	Decreased educational attainment in the family *p* = 0.002	Decreased educational attainment in the family *p* = 0.006
**MBzP**	Younger > older *p* = 0.04	*p* > 0.05
**MEOHP**	Urban > rural *p* = 0.03	*p* > 0.05

* Results where significant association found in mother or child (*p* < 0.05).

**Table 5:** Association between phthalate exposure and environmental variables.

Compound	Irish Children	Irish Mothers
**MEP**	PCP *p* = 0.02 ↑↑	Consumption of meat *p* = 0.02 *↓↑* Convenience foods in last 24 h *p* = 0.02 ↑↑
**MnBP**	*p* > 0.05	Plastic gloves *p* = 0.03 ↑↑
**MiBP**	PVC *p* = 0.007 ↑↑	Consumption of fast food *p* = 0.02 ↑↑
**MBzP**	Cheese *p* = 0.007 ↓↑	None
**MEHP**	Chewing gum *p* = 0.03 ↑↑	*p* = 0.26
**MEHHP**	Chewing gum *p* = 0.02 ↑↑	*p* = 0.52
MEOHP	Chewing gum *p* = 0.005 ↑↑	*p* = 0.44

Results where significant association found in mother or child (*p* < 0.05); Key to symbols: ↓↑ = inverse association ↑↑ = positive association.

## Supplementary Materials

The following are available online at www.mdpi.com/1660-4601/14/12/1456/s1, DEMOCOPHES Basic Questionnaire.
