# Supplementary material for: Urinary Phthalate Concentrations in Mothers and Their Children in Ireland: Results of the DEMOCOPHES Human Biomonitoring Study
Source: Int J Environ Res Public Health. 2017 Nov 25;14(12):1456. doi: 10.3390/ijerph14121456 (PMC5750875; doi:10.3390/ijerph14121456)
Supplement: Supplementary file 1 [file ijerph-14-01456-s001.pdf]

DEMOCOPHES

Basic Questionnaire

ID no. of child	_ _ _ _ _ _
ID no. of mother	_ _ _ _ _ _
Date *(of interview)*	_ _ _ _.201_
ID no. of interviewer	_ _
Signature Interviewer	

A. Residential environment and residence

1.	When was the building constructed you are currently living in?		
	year	_ _ _ _	
	don't know	○	

2.	How many square metres of living space does your home have?		*m² without decimals*
	m^2	_ _ _	

3.	In what area is your flat/house located?		*One tick only! The predominant situation around the home.*
	city centre	○	
	near city centre	○	
	suburb	○	
	industrial	○	
	rural /village	○	
	don't know	○	

4.	Is there any of the following within a 50 m radius around your home?			*Please tick in each row! If the interviewee cannot give an answer, you have to find out this information by walking around after the interview is completed.*
		yes	no	
	A. a metalworking business (e.g. a locksmithery, steel constructions)	○	○	
	B. a scrap yard	○	○	
	C. a waste incineration plant	○	○	
	D. a commercial solvent user (e.g. a painting business)	○	○	
	E. a landfill site	○	○	

5.	**What is the main type of heating in your home?**		*One tick only!*
	A. individual stove/heater in each room	○	
	B. heating system covering one floor/one flat	○	
	C. central heating (one boiler in the building)	○	
	D. district heating (no boiler in the building)	○	
	E. don't know	○	

6.	**What fuel or energy source do you mainly use for cooking? What is the main fuel or energy source for heating?**			*Please tick in each column!*
		cooking	heating	
	A. oil	○	○	
	B. gas	○	○	
	C. coal, charcoal, wood	○	○	
	D. electric power	○	○	
	E. geothermal energy		○	
	F. solar energy		○	
	G. other	○	○	
	which one?	....		
	H. don't know	○	○	

7.	**Are there any <u>additional</u> stoves/heaters used in your home that are fired with wood/ coal?** *This would cover tile stoves, bathing furnaces, multi-fuel burners and open fireplaces.*		
	yes	○	
	no	○	

8.	**Has your flat/house been redecorated within the last year?**		*Redecoration: inter alia, painting the walls, adding a new floor or new tiles.*
	no	○	
	yes	○	
	how many months ago?	_ _	

9.	**Has your flat/house been renovated within the last two years?**		*Renovation: inter alia, new windows, separation of a room by a wall, new pipes, convert the attic.*
	no	○	
	yes	○	
	how many months ago?	_ _	

10.	**Do you have PVC flooring in your flat/house?** *note: we don't mean linoleum!*		
	no	○	
	yes	○	
	how many m²?	_ _ _	
	don't know	○	

11.	**Do you have PVC wall paper in your flat/house?**		
	no	○	
	yes	○	
	how many m²?	_ _ _	
	Don't know	○	

B. Nutrition

1.	**What is your <u>main</u> source of water for drinking?** *Please also consider the water used to prepare coffee, tea, juices, etc.*		*One tick only!*
	A. public water supply	○	
	B. commercial producers (bottle, plastic vat/container, tetra pack, tins)	○	
	C. own well/ private water supply	○	
	D. don't know	○	

2.	**What is your <u>main</u> source of water for cooking?**		*One tick only!*
	A. public water supply	○	
	B. commercial producers (bottle, plastic vat/container, tetra pack, tins)	○	
	C. own well/private water supply	○	
	D. don't know	○	

3.	**How often do you drink alcohol? Base your answer on the last year.**								*A Unit of beer usually contains ½ pint, a glass of wine is 2units and a standard spirits is 2 units.*<br><br>*Please tick in each row!*
		≥1 Units a day	5-6 Units a week	2-4 Units a week	1 unit a week	1-3 Units a month	<1 unit a month	never	
	A. Beer	○	○	○	○	○	○	○	
	B. Wine, fruit wine, sparkling wine, cider	○	○	○	○	○	○	○	
	C. Spirits	○	○	○	○	○	○	○	

4.	**Ms. *[name]*, how often did you eat the following foods in the last 4 weeks?**							*Please tick in each row!*
		several times a day	daily	several times a week	1x a week	2-3x a month	1x a month	almost never	
	A. rice	○	○	○	○	○	○	○	
	B. meat/ cold meat (*ham, cut*)	○	○	○	○	○	○	○	
	C. offal (*liver, kidneys, sweetbread etc.)*	○	○	○	○	○	○	○	
	D. game	○	○	○	○	○	○	○	
	E. wild mushrooms	○	○	○	○	○	○	○	
	F. hazelnut spread	○	○	○	○	○	○	○	
	G. convenience food/ fast food *(frozen pizza, other already prepared meals)*	○	○	○	○	○	○	○	
	H. milk	○	○	○	○	○	○	○	
	I. cheese	○	○	○	○	○	○	○	
	J. cereals	○	○	○	○	○	○	○	
	K. chocolate	○	○	○	○	○	○	○	
	L. ice cream	○	○	○	○	○	○	○	
	M. local food *(home-grown fruit/ vegetables (incl. potatoes) from your own/ relative's/ friend's garden or from a farm nearby)*	○	○	○	○	○	○	○	
	N. dishes served in a canteen	○	○	○	○	○	○	○	
	O. chewing gum	○	○	○	○	○	○	○	

5.	How often did your child *[name]* eat the following foods in the last 4 weeks?								*Please tick in each row!*
		several times a day	daily	several times a week	1x a week	2-3x a month	1x a month	almost never	
	A. rice	○	○	○	○	○	○	○	
	B. meat/ cold meat *(ham, cut)*	○	○	○	○	○	○	○	
	C. offal (*liver, kidneys, sweetbread etc.)*	○	○	○	○	○	○	○	
	D. game	○	○	○	○	○	○	○	
	E. wild mushrooms	○	○	○	○	○	○	○	
	F. hazelnut spread	○	○	○	○	○	○	○	
	G. convenience food/ fast food *(frozen pizza, other already prepared meals)*	○	○	○	○	○	○	○	
	H. milk	○	○	○	○	○	○	○	
	I. cheese	○	○	○	○	○	○	○	
	J. cereals	○	○	○	○	○	○	○	
	K. chocolate	○	○	○	○	○	○	○	
	L. ice cream	○	○	○	○	○	○	○	
	M. local food *(home-grown fruit/ vegetables (incl. potatoes) from your own/ relative's/ friend's garden or from a farm nearby)*	○	○	○	○	○	○	○	
	N. dishes served in a canteen	○	○	○	○	○	○	○	
	O. chewing gum	○	○	○	○	○	○	○	

6.	**Ms.** ***[name]*****, how often did you eat fish/fish products in the last 4 weeks?**								
		several times a day	daily	several times a week	1x a week	2-3x a month	1x a month	almost never	
	A. fish/shellfish *(this includes tuna in a salad or on a sandwich/ pizza, prawn cocktail, sea weed etc.)*	○	○	○	○	○	○	○	

7.	**Ms.** ***[name]*****, how often did you eat the following <u>fish products</u> in the last 4 weeks?**								*Please tick in each row!*
		several times a day	daily	several times a week	1x a week	2-3x a month	1x a month	almost never	
	B. sea fish	○	○	○	○	○	○	○	
	C. shellfish	○	○	○	○	○	○	○	
	D. freshwater fish	○	○	○	○	○	○	○	
	E. other sea products (e.g. sea weed)	○	○	○	○	○	○	○	

8.	**How often did your child** ***[name]*** **eat fish/fish products in the last 4 weeks?**								
		several times a day	daily	several times a week	1x a week	2-3x a month	1x a month	almost never	
	A. fish/shellfish *(this includes tuna in a salad or on a sandwich/ pizza, prawn cocktail, sea weed etc.)*	○	○	○	○	○	○	○	

9.	**How often did your child** ***[name]*** **eat the following <u>fish products</u> in the last 4 weeks?**								*Please tick in each row!*
		several times a day	daily	several times a week	1x a week	2-3x a month	1x a month	almost never	
	B. sea fish	○	○	○	○	○	○	○	
	C. shellfish	○	○	○	○	○	○	○	
	D. freshwater fish	○	○	○	○	○	○	○	
	E. other sea products (e.g. sea weed)	○	○	○	○	○	○	○	

C. Smoking behaviour

1.	Does anyone smoke in your flat/house indoors?		
	yes	○	
	no	○	*Please go to question 4!*

2.	How many people usually smoke in your flat/house indoors?		
	number of people	_ _	

3.	**And how many cigarettes/cigars/pipes per day are usually smoked in your flat/house indoors?** *Please give an estimate if you don't know exactly*		
	cigarettes/cigars/pipes per day	_ _ _	

4.	**Do you smoke, Ms.** *[name]***?**		
	yes, daily	○	
	yes, occasionally	○	
	no, I gave up smoking…	○	
	years ago	_ _	
	months ago	_ _	
	no, I have never smoked	○	*Please go to question 6!*

5.	**How much do you currently smoke per day on average?**		*Multiple answers possible*
	Number of		
	A. cigarettes	_ _	
	B. cigars/ cigarillos (small cigars)	_ _	
	C. pipes	_ _	

6.	**Does your child** *[name]* **smoke?**		
	yes, daily	○	
	yes, occasionally	○	
	no, he/she gave up smoking…	○	
	months ago	_ _	
	no, he/she has never smoked	○	
	don't know	○	

7.	**How often do visitors smoke in your home?** *For instance, when you host parties and other special occasions*		
	daily	○	
	4-6 times a week	○	
	2-3 times a week	○	
	once a week	○	
	less often	○	
	never	○	
	don't know	○	

8.	**How often are you exposed to tobacco smoke in indoor settings?**							*Please tick in each row!*
		daily	4-6x a week	2-3x a week	1x a week	less often	never	
	A. at home	○	○	○	○	○	○	
	B. at friends/ relatives/ neighbours	○	○	○	○	○	○	
	C. in restaurants, ice-cream parlours, pubs, discotheques, sports clubs, at events	○	○	○	○	○	○	
	D. at work	○	○	○	○	○	○	
	E. in car, bus, train and other means of transport	○	○	○	○	○	○	
	F. elsewhere: *(where?)* ……………………..	○	○	○	○	○	○	

9.	**How often is your child** *[name]* **exposed to tobacco smoke in indoor settings?**							*Please tick in each row!*
		daily	4-6x a week	2-3x a week	1x a week	less often	never	
	A. at home	○	○	○	○	○	○	
	B. at friends/ relatives/ neighbours	○	○	○	○	○	○	
	C. in restaurants, ice-cream parlours, pubs, discotheques, sports clubs, at events	○	○	○	○	○	○	
	D. at the parents' work place	○	○	○	○	○	○	
	E. in car, bus, train and other means of transport	○	○	○	○	○	○	
	F. elsewhere: *(where?)* ……………………..	○	○	○	○	○	○	

D. Exposure-relevant behaviour

1.	**Ms. *[name]*, how often do you use…?**					*Please tick in each row!*
		(almost) every day	about every second day	about 1 x a week	less often/never	
	A. make up	○	○	○	○	
	B. eye make up	○	○	○	○	
	C. shampoo	○	○	○	○	
	D. hair styling products	○	○	○	○	
	E. body lotion, creams (e.g. hand creams, lip balsam etc.)	○	○	○	○	
	F. fragrances (perfume, eau de toilette etc.)	○	○	○	○	
	G. deodorants	○	○	○	○	
	H. massage oil	○	○	○	○	
	I. nail polish	○	○	○	○	

2.	**How often does your child *[name]* use …?**					*Please tick in each row!*
		(almost) every day	about every second day	about 1 x a week	less often/never	
	A. make up	○	○	○	○	
	B. eye make up	○	○	○	○	
	C. shampoo	○	○	○	○	
	D. hair styling products	○	○	○	○	
	E. body lotion, creams (e.g. hand creams, lip balsam etc.)	○	○	○	○	
	F. fragrances (perfume, eau de toilette etc.)	○	○	○	○	
	G. deodorants	○	○	○	○	
	H. massage oil	○	○	○	○	
	I. nail polish	○	○	○	○	

3.	**Have you ever used treatments for skin bleaching?**		
	no	○	*Please enter the number of years or months that passed since the last treatment.*
	yes	○	
	How long ago?		
	years ago	_ _	
	months ago	_ _	
	name of product (if known):	…………………………….	

4.	**Has your child *[name]* ever used treatments for skin bleaching?**		
	no	○	*Please enter the number of years or months that passed since the last treatment.*
	yes	○	
	How long ago?		
	years ago	_ _	
	months ago	_ _	
	name of product (if known):	……………………………...	

5.	**Do you have teeth with amalgam fillings, Ms. *[name]*?**		
	yes	○	
	how many?	_ _	
	no amalgam fillings	○	
	don't know	○	

6.	**Does your child *[name]* have teeth with amalgam fillings?**		
	yes	○	
	how many?	_ _	
	no amalgam fillings	○	
	don't know	○	

7.	**How much time do you, Ms.** *[name]*, **spend in a car per day on average?** *This refers to all cars you use privately and/or in your job at workdays and at the weekend.*		
	_ _ hours	_ _ minutes	*if < 1 hour please fill in zero=0 and specify minutes*

8.	**How much time does your child** *[name]* **spend in a car per day on average?** *This refers to all cars you use privately and/or in your job at workdays and at the weekend.*		
	_ _ hours	_ _ minutes	*if < 1 hour please fill in zero=0 and specify minutes*

9.	**How old is the car which you use mainly, Ms.** *[name]*?		
	years	_ _	
	months	_ _	

10.	**How old is the car your child** *[name]* **spends the most time in?**		
	years	_ _	
	months	_ _	

11.	**Has a clinical thermometer containing mercury** *(silver-coloured fluid)* **ever been broken in your home?**		
	yes	○	
	How long ago?	_ _ years _ _ months _ _ days	
	no	○	
	don't know	○	

12.	**Has an energy-saving lamp ever been broken in your home?**		
	yes	○	
	How long ago?	_ _ years _ _ months _ _ days	
	no	○	
	don't know	○	

13.	**Does anybody work with a soldering metal in your home?** *This might be as part of DIY and hobby activities, for instance.*		
	yes	○	
	no	○	

14.	**Did anybody work regularly with the following materials in your home in the last four weeks?** *This might be as part of do it yourself (DIY) and hobby activities, for instance.*			*Please tick in each row!*
		yes	No	
	A. metals	○	○	
	B. paints/ coatings	○	○	
	C. lubricants	○	○	

15.	**How often did you wear rubber-like plastic gloves (not latex) in the last week?**		
	daily	○	
	less than daily	○	
	never	○	

16.	**How often did your child** *[name]* **play with toys consisting of or containing rubber-like plastic in the last week?** *Please also think of toys your child plays with outside your home.*		
	daily	○	
	less than daily	○	
	never	○	
	don’t know	○	

17.	**How much time per day does your child spend outdoors? Please think of a normal school day during the last 12 months.**		
	in summertime	_ _ hours _ _ minutes	
	in wintertime	_ _ hours _ _ minutes	

E. Occupation

1.	What kind of professional/vocational training do you have?	
	…………………………………………………………..	

2.	Which is your main occupation at present?		
	…………………………………………………………		*If answer A: please go to section F!*
	A. unemployed/ housewife/ out of work/ student	○	
	B. in training	○	*If answer B: please proceed with question 3.*

3.	To which industrial sector does this occupation belong?		*Don't read out the possible options, only tick them if named!* *If the interviewee cannot answer properly, give support according to the present occupation.*
	A. Agriculture, forestry and fishing	○	
	B. Mining and quarrying	○	
	C. Manufacturing	○	
	D. Electricity, gas, steam and air conditioning supply	○	
	E. Water supply, sewerage, waste management and remediation activities	○	
	F. Construction	○	
	G. Wholesale and retail trade, repair of motor vehicles, motorcycles	○	
	H. Transportation and storage	○	
	I. Accommodation and food service activities	○	
	J. Information and communication	○	
	K. Financial and insurance activities	○	
	L. Real estate activities	○	
	M. Professional, scientific and technical activities	○	
	N. Administrative and support service	○	
	O. Public administration and defence, compulsory social security	○	
	P. Education	○	
	Q. Human health and social work activities	○	
	R. RTS, entertainment and recreation	○	
	S. Other service activities	○	

4.	Please specify this sector!	
	………………………………………………………………….... …………………………………………………………………....	

5.	**When did you start this job?**		
	year	_ _ _ _	

6.	**What is your work environment in this job most of the time?**		
	A. offices, schools, kindergartens, libraries, etc.	○	
	B. factory buildings, warehouses etc.	○	
	C. shops, canteens, restaurants, etc.	○	
	D. hospitals, doctor's surgery, labs	○	
	E. indoor construction sites	○	
	F. outdoor construction sites	○	
	G. traffic (car, train, pedestrian)	○	
	H. forests, parks, fields, barns etc.	○	

7.	**Do you come into contact with the following substances on this job:**			*Please tick in each row.*
		yes	No	
	A. metallic dust	○	○	
	B. oil	○	○	
	C. pharmaceuticals	○	○	
	D. paints/ coatings	○	○	
	E. solvents	○	○	
	F. plasticiser	○	○	
	G. mercury	○	○	
	H. cadmium	○	○	
	I. other metals	○	○	
	Which ones?	…………………………………		

8.	**Do you come into contact with hazardous materials, hazardous waste or other special chemicals?**		
	no	○	
	don't know	○	
	yes	○	
	which ones?	……………………………………………………	

F. Socio-Demography

1.	**Are you living alone with your child / children, Ms.** *[name]***?**		
	yes	○	
	no	○	

2.	**Where were you born?** *Please answer for you and father/spouse/partner if possible!*				
		child surveyed	mother	father/spouse/partner	
	in *[member state]*	○	○	○	
	in another country	○	○	○	
	which one?	…………….	…………….	…………………………	

3.	**Since when have you lived in** *[member state]***?** *Please answer for you and father/spouse/partner if possible!*				
		child surveyed	mother	father/spouse/partner	
	since birth	○	○	○	
	since *[year]*	_ _ _ _	_ _ _ _	_ _ _ _	

4.	**Which languages do you speak at home?**		
	language of member state	○	
	other language(s)	○	
	which one(s)?	…………………..	

5.	**Please tell us for ALL of your household members: gender, age and smoking status.**					
		gender		age	smoker	
		m	f		yes	no
	mother			_ _	○	○
	child surveyed	○	○	_ _	○	○
	1. other person	○	○	_ _	○	○
	2. other person	○	○	_ _	○	○
	3. other person	○	○	_ _	○	○
	4. other person	○	○	_ _	○	○
	5. other person	○	○	_ _	○	○
	6. other person	○	○	_ _	○	○
	7. other person	○	○	_ _	○	○
	8. other person	○	○	_ _	○	○

6.	**What education do you have, Ms.** *[name]***?** *Please answer for you and father/spouse/partner if possible!*			*Please tick in each column but only once per column!*<br><br>*Categories should be adapted according to member state.*
		mother	father/spouse/partner	
	A. no formal education	○	○	
	B. primary education	○	○	
	C. lower secondary education, Up to Junior/Inter Cert	○	○	
	D. (upper) secondary education Up to Leaving Cert	○	○	
	E. Post Leaving Cert Courses, FAS, FETAC, etc	○	○	
	F. Third Level, University/college, Diploma, Batchelors degree	○	○	
	G. Masters, Phd, Research	○	○	
	H. don’t know		○	

7.	**What is your current labour status?** *Please answer for you and father/spouse/partner if possible!*			*Please tick in each column!*
		mother	father/spouse/ partner	
	A. Carrying out a job or profession, including unpaid work for a family business or holding, including an apprenticeship or paid traineeship, etc.	○	○	
	Full time	○	○	
	Part time	○	○	
	B. Unemployed	○	○	
	C. Pupil, student, further training, unpaid work experience	○	○	
	D. in retirement or early retirement or has given up business	○	○	
	E. permanently disabled	○	○	
	F. in compulsory military or community service	○	○	
	G. fulfilling domestic tasks	○	○	
	H. other inactive person	○	○	

8.	**Which position do you presently hold?** *Please answer for you and father/spouse/partner if possible!*			*Please tick in each column!*
		mother	father/spouse/partner	
	A. Manager	○	○	
	B. Professional	○	○	
	C. Technician or associate professional	○	○	
	D. Clerical support worker	○	○	
	E. Service or sales worker	○	○	
	F. Skilled agricultural, forestry or fishery worker	○	○	
	G. Craft and related trade worker	○	○	
	H. Plant or machine operator or assembler	○	○	
	I. Elementary occupation	○	○	
	J. Armed forces occupation	○	○	

9.	**Perhaps you can indicate the category your household's MONTHLY DISPOSABLE NET INCOME belongs to. That is the gross income of all household members minus regular taxes on wealth, inter-household cash transfers paid, tax on income and social insurance contributions?**		
	< 50 % of the MS mean net income	<2000	○
	50 % - < 60 % of the MS mean net income	2000- <2250	○
	60 % - < 75 % of the MS mean net income	2250- <2750	○
	75 % - < 90 % of the MS mean net income	2750 - <3500	○
	90 % - < 115% of the MS mean net income	3500 - <4500	○
	115 % - < 150 % of the MS mean net income	4500 - <5500	○
	150 % - < 200 % of the MS mean net income	5500 - <7500	○
	> 200 % of the MS mean net income	> 7500	○

Note: The average monthly disposable household income is 3830 (Cental Statistics Office – Ireland) 2009
